# Pain Perception and the Opioid Receptor Delta 1

**DOI:** 10.7759/cureus.2149

**Published:** 2018-02-04

**Authors:** Adam T Hilgemeier, David M Serna, Tarak P Patel, Kerry Anne Rambaran, Zubair M Amin, Jie An, Saeed K Alzghari

**Affiliations:** 1 School of Pharmacy, Texas Tech University Health Sciences Center; 2 Department of Clinical Sciences, Keck Graduate Institute; 3 Thomas J. Long School of Pharmacy & Health Sciences, University of the Pacific; 4 Gulfstream Genomics, Gulfstream Diagnostics

**Keywords:** oprd1, pain, opioids, genetics, pharmacogenetics

## Abstract

Genetic factors play an integral role in the perception of pain, and studies have only recently begun to explore the degree to which these factors affect clinical decisions. The process of prescribing opioids is greatly influenced by an individual's pain perception, which can vary based on several factors including genetic variation. Opioid receptor delta 1 (OPRD1) plays a significant role in the perception of both pain and its relief via opioids, and it shows significant variability between individuals. Herein, we discuss the nature of the OPRD1 receptor and the value of further research into its effects, particularly in the realm of pain management.

## Editorial

Pain has been dubbed the fifth vital sign since 1996 and is attributed to approximately one-fifth of physician visits [1]. Moreover, an estimated 20% of adults suffer from chronic pain globally, and a further 10% are diagnosed every year [1]. Opioid prescriptions play a significant role in pain management; however, issues concerning the genetic variability in both pain perception and response to opioids need to be further elucidated.

The analgesic effect of opioids is driven by opioid receptors, which function endogenously to dampen pain signals and have several subtypes: delta, kappa, mu, and zeta [2]. Each receptor subtype governs responses to different stimuli (such as heat or pressure) and origin (such as visceral or peripheral). The opioid receptor delta 1 (OPRD1) shows significant variation between individuals and may modulate several physiological effects of opioids including analgesia, respiratory depression, and physical dependence [2-4]. Most of the data concerning the function of OPRD1 derives from animal studies and suggests a correlation between delta receptors and mechanical nociception. However, recent studies in individuals treated with opioids have shown that single nucleotide polymorphisms (SNPs) within the OPRD1 receptor (including rs2234918, rs419335, and rs533123) result in differences to pain response as assessed by visceral heat and muscle pressure tests [3-4].

In addition to its regulatory roles with visceral and mechanical nociception, recent findings suggest that OPRD1 plays a complementary role in the process of pain management. Specifically, heteromer receptors (mu-delta) have been targeted to elicit a prolonged nociceptive response with morphine use [2]. By utilizing a combination ligand that targets the delta receptor along with the mu receptor, the normal effects of morphine are further potentiated with the activation of the delta receptor. The mu-delta heteromer response may have application in reducing opioid tolerance than single-receptor agonists [2].

Recent studies have shown new applications of OPRD1 in pain management. The nature of pain associated with osteoarthritis, the world's most common joint disease, may be modulated in large part by OPRD1; a 2017 study regarding SNP variations in osteoarthritis patients demonstrated a significant increase in pain detection threshold to contact heat stimulation in patients carrying the OPRD1 rs2234918C allele [5]. A decrease in pain threshold in individuals with osteoarthritis could lead to significant decreases in quality of life as well as a reduction in activities of daily living, negatively impacting outcomes in these patients. A significant difference in pain perception has also been linked to gender, as shown in a recent study where males showed an increased response to thermal stimulation [4]. This may be due to inter-individual variability or indicative of demographic response variations. In either case, there are more questions than answers regarding the role of OPRD1 and pain perception.

Further studies are required to define the impact of genetic expression of OPRD1 and pain perception. Currently, the literature suggests that patients with osteoarthritis may benefit from further exploration into the processes influenced by OPRD1 variants. A robust study will stratify by demographics including sex and race since recent findings suggest that demographic variation may play a significant role in pain perception. Additionally, the degree of response variation in heteromers targeting mu- and delta-opioid receptors must be further examined to elucidate the synergistic phenomena observed by simultaneous mu and delta ligands. Further research into the pharmacogenetics of the OPRD1 receptor is a potential avenue to understanding its role in pain management and fits within the scope of the United States' battle in curtailing the current opioid crisis. Lastly, other types of pain and its relationship to OPRD1 are worth exploring further.

In conclusion, the OPRD1 receptor may be involved in pain perception and management, but the extent of its role in clinical decision making is underexplored. Demographics, disease states, and the synergistic role of heteromer ligands must be further explored for a complete understanding of pharmacological pain management.
